# Emergency Department Visits for Sexual Assault by Emerging Adults: Is Alcohol a Factor?

**DOI:** 10.5811/westjem.2018.6.38219

**Published:** 2018-07-26

**Authors:** Allison Tadros, Melinda J. Sharon, Shelley M. Hoffman, Danielle M. Davidov

**Affiliations:** * West Virginia University School of Medicine, Department of Emergency Medicine, Morgantown, West Virginia; † West Virginia University School of Public Health, School of Medicine, Department of Social and Behavioral Sciences, Morgantown, West Virginia

## Abstract

**Introduction:**

Emerging adults (18–25 years of age) are at increased risk for sexual assault. There is little Emergency Department (ED) data on sexual assaults that involve alcohol among this population. The purpose of this study was to analyze ED visits for sexual assault and determine if alcohol consumption by the patient was noted.

**Methods:**

This study was a retrospective chart review of patients aged 18–25 presenting to an ED in a college town over a four-year period. Extracted variables included age, gender, delay in seeking care, sexual assault nurse examiner (SANE) evaluation, and alcohol consumption by the patient. For analysis of alcohol use, cases were categorized as ages < 21 and ≥ 21.

**Results:**

There were 118 patients who presented to the ED from 2012 to 2015. The mean age of the cohort was 20 years, and almost 70% of visits were among those < 21. Of those aged < 21, 74% reported alcohol consumption, in contrast to 48% of those ≥ 21 (p = 0.055). Of those reporting alcohol use, 36% were evaluated on the day of the assault compared to 61% of those not reporting alcohol (p=0.035).

**Conclusion:**

This study found that ED visits for sexual assault in emerging adults were more common in younger patients. Alcohol use occurred more frequently with patients under the legal drinking age, and presentation was also more likely to be delayed. The relationship between sexual assault and alcohol use should underscore primary prevention efforts in emerging adult populations.

## INTRODUCTION

Sexual assault is a complex public health and medical problem. The vast majority of sexual assaults against females occur before age 25.^1^ Approximately 38% of victims of completed rape, which includes forced penetration and completed alcohol- or drug-facilitated rape, first experience this form of sexual assault between the ages of 18 and 24.^1^ The term “emerging adulthood” has been recently used to describe the developmental period between ages 18 and 25.^2^ This phase is characterized by significant life transitions such as entry into the workforce and/or college attendance as well as sharp increases in experimentation and unsafe behavior, making this age group vulnerable to violence and substance use.^3^–^5^ Because over one-third of emerging adults in the United States (U.S.) attend college, the disproportionate impact of sexual assault among this population is a growing concern.^6^ Sexual assault on college campuses is an issue that has recently caught the attention of national leaders in the U.S., as evidenced by the creation of the “It’s On Us” and White House Task Force to Protect Students from Sexual Assault initiatives.^7^,^8^ Studies have found that as many as 1 in 5 women on college campuses experience sexual assault, and a high percentage of these are facilitated by alcohol.^9^ Few studies have focused on the relationship between alcohol and sexual assault among emerging adult populations that do not attend college.^8^

Most prior research has focused on the relationship between sexual assault and alcohol consumption by the perpetrator, yet recent studies estimate that up to 70% of young adult victims report alcohol consumption prior to the incident.^10^–^14^ Being under the influence of alcohol can impair both parties’ abilities to give and recognize active consent to engage in sexual activity.^15^ Furthermore, individuals who were intoxicated during an incident of sexual assault were less likely to report the incident because they were “unclear if crime had been committed” or “didn’t think incident was serious enough” to report.^16^–^19^ Alcohol use by the victim might also have an impact on help and healthcare seeking, with individuals using alcohol at the time of the assault being less likely to call law enforcement or seek medical treatment.^17^ Although several studies have reported that alcohol intoxication was associated with less frequent reporting to law enforcement and medical evaluation, little research has examined delayed presentation to the emergency department (ED) after assault.^19^ Delayed care may significantly impact patients’ abilities to have forensic evidence collected during sexual assault nurse examiner (SANE) exams.^20^

Most previous research on the relationship between sexual assault and alcohol consumption among emerging adults: 1) aims to establish directionality and causal explanations (i.e. how does alcohol influence the likelihood sexual assault occurrence?); 2) utilizes self-report/self-administered surveys; and/or 3) focuses on college-attending samples.^15^, ^21^–^24^ However, fewer studies have utilized healthcare data to describe characteristics of medical treatment received by emerging adults who have experienced sexual assault and report alcohol use at the time of victimization. The ED is a frequent point of entry for patients experiencing sexual assault. EDs play a critical role by providing immediate care, facilitating forensic data collection through the use of SANE exams, and connecting patients with community resources such as sexual assault advocacy services and counselors.^25^ This makes medical record data a valuable, yet underutilized resource for gleaning information on characteristics associated with sexual assault. To our knowledge, no prior study has specifically examined patient- and visit-level characteristics of emerging adults presenting to the ED following a report of sexual assault. The purpose of this study was to conduct a medical record review of patients between the ages of 18–25 who presented to our ED after sexual assault victimization. We sought to examine reported alcohol use at the time of the incident as well as various demographic and clinical characteristics among these patients.

**Population Health Research Capsule**

What do we already know?

The majority of sexual assaults occur before the age of 25 years. Patients commonly seek care following a sexual assault in the emergency department (ED).

What was the research question?

How commonly is alcohol use reported in young adults presenting to the ED following a sexual assault?

What was the major finding of the study?

Alcohol use at the time of the assault was reported in 60% of patients, and the majority of patients were under 21 years of age.

How does this improve public health?

Primary prevention efforts, including those on college campuses, should address and incorporate the relationship between sexual assault and alcohol use.

## METHODS

This was a retrospective medical record review of all patients ages 18–25 presenting to the ED of a tertiary care, academic hospital in the mid-Atlantic region of the U.S. between January 1, 2012 and December 31, 2015. The hospital resides in a college town where over half of the population is comprised of college students. The undergraduate student enrollment is approximately 22,500, of which 54% are male. The first author (AT) reviewed medical records for all patients with ICD-9-CM codes of E960.1 (rape) or V71.5 (observation following alleged rape or seduction) present in the discharge diagnosis during the study period. The following information was extracted from each relevant case: age, gender, delay in seeking care (patient not presenting the same day as the incident), evaluation by the SANE nurse, prophylaxis for human immunodeficiency virus (HIV), sexually transmitted infections (STI) and pregnancy, and if alcohol consumption by the patient was recorded in the medical record. Female patients with an intrauterine device, implanted contraception device, or taking oral contraceptives as prescribed were considered to be on reliable contraception. Descriptive statistics (i.e. frequencies and percentages) were used to describe all study variables. Chi-square tests were used to examine bivariate associations between age (grouped as <21 and ≥ 21) and alcohol use as well as delay in presentation to the ED (<24 hours vs. ≥ 24 hours) and alcohol use. Statistical significance was set to p <0.05 for all analyses. Data was analyzed using SPSS 22.0. This study was approved by our institution’s Institutional Review Board.

## RESULTS

**Figure 1:**
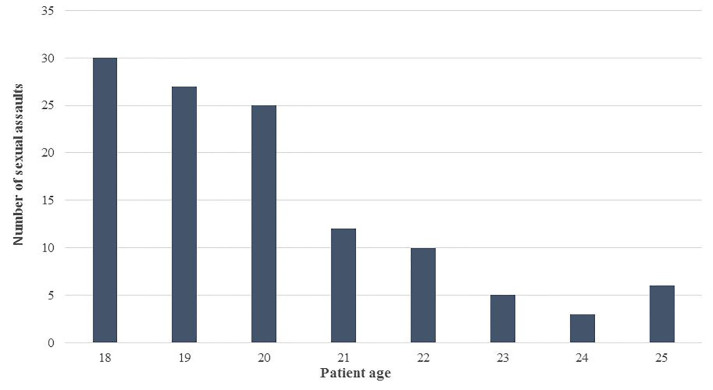
Number of sexual assaults by age among emerging adults.

**Figure 2:**
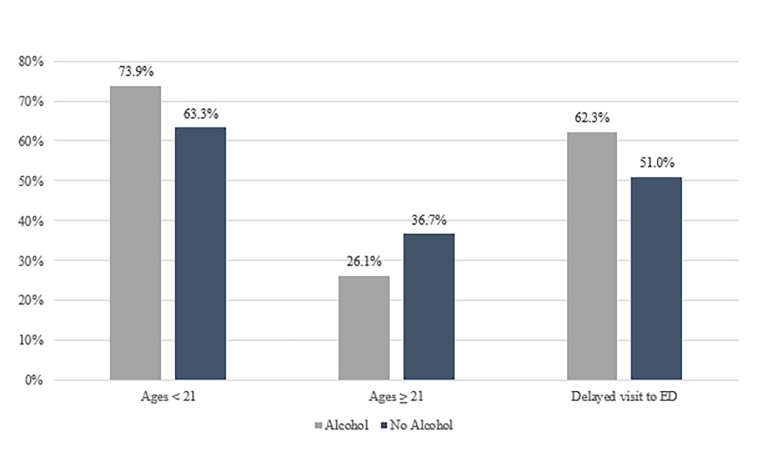
Percentage of sexual assault cases with reported alcohol use by age group among emerging adults.

**Figure 3:**
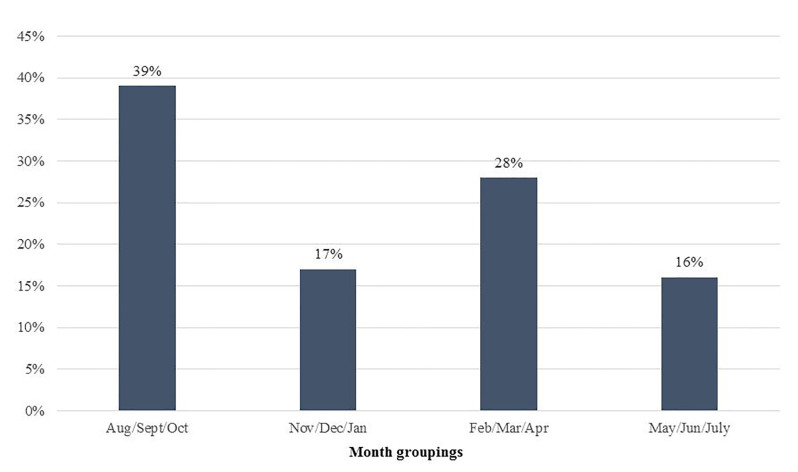
Percentage of sexual assault cases presenting to the ED by month groupings among emerging adults.

There were a total of 121 emerging adult patients presenting for evaluation after sexual assault during the study period, 98% (n=118) of which were female. To minimize the risk of inadvertent disclosure of private information, no further characteristics of the three male patients are reported here. Thus, the subsequent findings presented reflect data from the 118 female patients. Almost 70% (n=82) were 21 years or younger, with a mean age of 20 (Figure 1). Other demographic information (e.g. race, socioeconomic status) is not routinely recorded in our electronic medical record and was unavailable for inclusion in our analyses. Almost 60% (n=69) of cases involved reported alcohol use by the patient. Of these, 74% were under age 21 and 26% were age 21 and over (*p* < 0.0002; Figure 2). Significantly more patients under 21 reported alcohol use than not (73.9% vs. 63.3%, *p* < 0.0018). A significantly greater proportion of patients reporting alcohol use at the time of the incident had a delay in presentation (of at least 24 hours) to the ED compared with those not disclosing alcohol use (62.3% vs. 37.7%, *p* < 0.0038). The number of patients seeking care for sexual assault also differed by the time of year, with almost 40% of visits occurring during late summer/early fall (Figure 3). Fewer visits occurred in winter and summer months. The table presents various clinical characteristics associated with ED visits for patients presenting after sexual assault. Prophylaxis for gonorrhea and chlamydia was accepted by 85.6% of patients. In contrast, less than a quarter of patients accepted HIV prophylaxis. Of those not already on reliable contraception, 83% accepted pregnancy prevention medication. An examination by a SANE nurse was accepted by 84% of patients.

## DISCUSSION

Alcohol use was prevalent in our sample of emerging adult patients presenting to the ED after sexual assault victimization, especially among those under 21 years of age. College students and other young adults—many of whom are under the legal drinking age of 21 in the U.S.—frequently participate in the practice of binge drinking (defined as 4 or more drinks an hour for women and 5 or more an hour for men), making them particularly susceptible to sexual assault.^26^,^27^ This finding is supported by previous research; a study by Lawyer and colleagues examining forcible, drug-facilitated, and incapacitated sexual assault and rape among undergraduate women found the average age among assault victims to be 19.^21^

In our study, alcohol use was also associated with a delay in presentation for ED care of at least 24 hours. Alcohol use may lead to delays in seeking healthcare after an assault due to incapacitation at the time of the incident or less certainty over what transpired during the assault due to the effects of alcohol. A seasonal variation in the number of sexual assaults presenting to the ED was observed, with the highest number occurring in the first three months of the academic year (August, September, and October). These months coincide with an influx of approximately 5,000 incoming freshmen as well as the beginning of football season, fraternity and sorority events, and many other on- and off-campus social gatherings. In fact, many notes in the patients’ medical records made specific mention of these types of events. Additionally, academic loads are often lighter at the beginning of the semester, giving students more free time to attend social events and engage in binge drinking behavior. College freshman new to campus may be particularly vulnerable to the effects of alcohol and may not yet have an established, trusted social network. Not surprisingly, the lowest number of assaults occurred during the months when students would be on holiday and summer breaks. Administrators and others involved in the education and prevention of sexual assaults should be aware of these variations.

A large portion of our population did elect to have SANE evaluations, emergency contraception, and prophylaxis for Gonorrhea and Chlamydia. Prophylaxis for pregnancy, Gonorrhea, and Chlamydia can be completed while in the ED. Lower rates of HIV prophylaxis were likely related to side effects, length of treatment, cost, and the lower prevalence of the disease in our catchment area.^28^

Given that emerging adulthood is a time when young persons are at increased risk for sexual assault and are also likely to engage in risky behaviors, such as binge drinking, sexual assault prevention efforts should begin early and integrate information about binge drinking and alcohol-related sexual assault into their curricula.^30^ Ideally this education would occur before emerging adults reach college age, as some individuals do not seek higher education but still may be vulnerable. Our data also support the notion that prevention efforts should occur prior to college, as the majority of sexual assaults in this study occurred during the beginning of the academic year among younger (>21 years of age) patients. Programs to encourage responsible drinking should also have a focus on alcohol’s role in sexual assault, and emphasize that alcohol impairs a partner’s ability to consent to sexual activity.^30^ In a survey of college students, over 40% of respondents believed a woman was responsible for the rape if she was intoxicated at the time.^31^ This misconception may add to feelings of guilt by the victim. Widely promoted risk reduction tactics to reduce the likelihood of sexual assault while engaging in drinking behavior include protecting drinks from possible alteration, staying in groups, being aware of alcohol limits, and not accepting rides from or going home with strangers. Still, prevention programs that primarily target the victim and emphasize awareness and sexual assault risk reduction alone have not demonstrated reductions in rates of sexual assault over time.^28^,^32^ Bystander-based prevention programs that focus on changes in social norms have shown promise for reducing sexual violence.^23^, ^33^–^35^

## LIMITATIONS

As many women do not report a sexual assault after it occurs, this study likely represents only a fraction of the total number of sexual assaults occurring in our community.^29^ In addition, because this study included all patients aged 18–25 presenting for evaluation of sexual assault, we were not able to differentiate which patients were college students and which were other young adults in the community. This may limit the applicability of our data to other student populations. Patients may have also reported to other health care settings, such as student health services. However, the protocol is to send patients to our ED from this clinic so this is not thought to be a common mechanism for missing patients.

This study was conducted in a retrospective fashion, and therefore, it was at the discretion of the provider as to whether or not they obtained and recorded information on the victim’s use of alcohol at the time of the incident. In addition, the patient may have intentionally not mentioned that they were using alcohol, especially as most of our victims were under the legal drinking age. Therefore, it is likely that alcohol consumption at the time of the sexual assault was even higher than what is represented in our data. Furthermore, lack of information about ethnicity, socioeconomic status, and other important demographic variables is a known limitation of using medical record data. Thus, we were unable to extract these data for our study. The characteristics of the three male patients included in our sample are not presented here, due to privacy and HIPAA concerns. Healthcare data on males receiving medical care after sexual assault is scarce and is an important area for future research.

## CONCLUSION

This study found that ED visits for sexual assault in emerging adults were more common in younger patients. Alcohol use occurred more frequently with patients under the legal drinking age; among this group, ED presentation was also more likely to be delayed. The relationship between sexual assault and alcohol use should underscore primary prevention efforts in emerging adult populations. Primary prevention efforts, including those on college campuses where a large portion of this population is present, should address and incorporate the relationship between sexual assault and alcohol use.

## Figures and Tables

**Table:** Clinical characteristics of sexual assault cases by age group among emerging adults.

Characteristics	<21 years n (%)	≥ 21 years n (%)	TOTAL n (%)
Total visits	82 (69.5)	36 (30.5)	118
Prophylaxis			
Gonorrhea/chlamydia	69 (84.1)	32 (88.9)	101 (85.6)
HIV	20 (24.4)	9 (25.0)	29 (24.6)
Plan B*	49 (83.1)	24 (82.8)	73 (83.0)
Alcohol involved	51 (62.2)	18 (50.0)	69 (58.5)
SANE evaluation	67 (81.7)	32 (88.9)	99 (83.9)

*HIV*, human immunodeficiency virus; *SANE*, sexual assault nurse examiner.

* Out of 88 eligible.
